# Evaluation of staining potential of Silver Diamine Fluoride, Potassium Iodide, Nanosilver Fluoride: an in vitro study

**DOI:** 10.1186/s12903-024-04370-y

**Published:** 2024-06-16

**Authors:** Büşra Karaduran, Sibel Çelik, Mehmet Koray Gök, Mine Koruyucu

**Affiliations:** 1 Department of Pediatric Dentistry, Faculty of Dentistry, Istanbul University, İstanbul, Türkiye; 2 Department of Chemical Engineering, Faculty of Engineering, Istanbul University-Cerrahpaşa, İstanbul, Türkiye

**Keywords:** Silver Diamine Fluoride, Potassium Iodide, Nanosilver Fluoride, Staining

## Abstract

**Background:**

The black staining effect of silver-containing solutions for use to arrest caries can have a negative aesthetic impact on children and parents. This study aims to assess the staining effects of Silver Diamine Fluoride/Potassium Iodide (SDF/KI), SDF and Nanosilver Fluoride (NSF).

**Materials and methods:**

Forty-four extracted carious primary molars were collected and randomly divided into four groups (*n* = 11). The carious tissue in all teeth was removed using a chemo-mechanical caries removal agent with an excavator. After caries removal in all groups, SDF, SDF/KI, and NSF were applied to the different groups, while no solution was applied to the control group. Subsequently, the teeth in all groups were restored with compomer. Color values L*, a* and b* were measured using a spectrophotometer at three time points: immediately after compomer restoration (T_0_), one week later (T_1_), and four week later (T_2_). Changes in brightness (ΔL) and color (ΔE) over time were calculated and comparisons among groups were made.

**Results:**

The SDF solution induced statistically significant black staining (*p* = 0.013) and a decrease in L* value (*p* < 0.001) on the compomer material compared to the other groups over time.

**Conclusions:**

It was observed that SDF/KI has the potential to reduce the black staining effect of SDF, though not entirely. Novel experimental solutions like NSF may offer an alternative to counteract the staining effect of SDF.

## Introduction

Dental caries is among the most prevelant chronic affecting children worldwide. Early Childhood Caries (ECC), defined as the presence of one or more cavities in the primary teeth of preschool-age children, as well as cavities that have been treated through extraction or restoration, is recognized as a significant public health concern. Furthermore, it is imperative to devise individualized strategies for the prevention and management of ECC, given its potential link to the development of cavities in adulthood [1]. Various materials have been developed for arresting the progression of dental caries and preventing its formation. Silver Diamine Fluoride (SDF), one of the products developed for this purpose, is a colorless and odorless solution that resembles water with an alkaline structure, containing silver, fluoride, and ammonium ions [2]. Its pH can vary within the range of 9 to 10, and it may cause metallic taste [3]. Based on a majority of clinical investigations, the biannual application of 38% SDF solution has yielded successful outcomes. However, its most significant disadvantages includes causing black staining in dental tissues, pulpal irritation, and oral soft tissue irritation [1]. It is stated that applying Potassium Iodide (KI) immediately after SDF helps reduce the risk of staining by binding with ionized silver [4]. A creamy white precipitate of silver iodide is formed as a result of the interaction with SDF. However, there are studies that indicate it may not be effective in preventing staining, or that it does not provide complete prevention, but rather reduces it to varying extents [5].

In order to develop new materials with strong antibacterial properties against the staining disadvantage of SDF, the use of nano silver particles has been considered. Due to their nano-scale dimensions and expansive surface area, they are capable of direct interaction with bacteria, resulting in bactericidal effect [6]. The inclusion of fluoride ions in its composition facilitates enamel remineralization and engenders a synergistic impact that reinforces bactericidal activity against cariogenic microorganisms [7]. The colloidal solution and varnish forms stand out as the types of NSF. It is mentioned that the synergistic effects of chitosan, sodium fluoride, and silver nanoparticles within the colloidal solution of NSF provide antibacterial and caries-preventing properties similar to SDF and do not lead to staining [8]. Hence, the objective of this in vitro study was to compare the extent of staining among SDF, SDF/KI, and NSF solutions on compomer. This in vitro experimental study was approved of the Clinical Research Ethical Board of Faculty of Dentistry, Istanbul University, Istanbul, Türkiye (No: 2022/21).

## Materials and methods

### Synthesis of Nanosilver Flouride

**Fig. 1:**
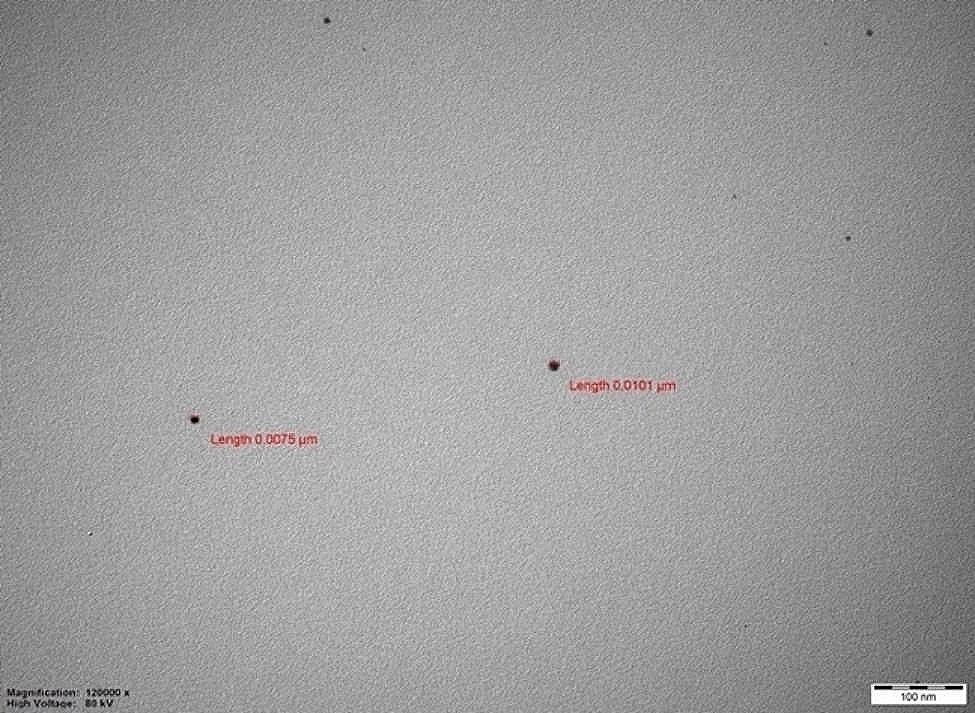
The TEM image of silver nanoparticles in NSF solution (1 μm = 1000 nm)

One g of chitosan (Sigma-Aldrich, Missouri, USA) was allowed to dissolve in 200 ml of deionized water containing 2% acetic acid (Sigma-Aldrich, Missouri, USA) overnight and it was subjected to centrifugation to eliminate insoluble components. Sixty ml of the resulting solution was introduced into the reaction bottle and positioned within ice bath arranged on mixer. Four ml of AgNO_3_ (0.012 mol/L) (Sigma-Aldrich, Missouri, USA) was executed, incorporating mixing speed of 500–600 rpm. This mixture was allowed to stir in the ice bath for 30 min. Afterward, dropwise addition of 1.5 ml of NaBH_4_ (0.8 mol/L) (Sigma-Aldrich, Missouri, USA) ensued, maintaining the mixture within the ice bath for another hour. NaF (0.0015 mol/L) (Sigma-Aldrich, Missouri, USA) was incorporated into the solution, which had reached room temperature. This solution was left to stir overnight under room temperature conditions in lightless environment to ensure stability. Transmission Electron Microscopy (TEM) (JEOL JEM-1011, Tokyo, Japan) was used to assess the dimensions of the silver nanoparticles. The analysis revealed that their size was less than 50 nm. The TEM image illustrating the evaluated particle size is depicted in Fig. 1.

### Tooth preparation and study setting

The G*Power software was used to detetmine sample number (V3.1.9.6). It was determined that there would be 11 samples in each group, resulting in a total of 44 samples across the four groups. Calculations were made with an effect size of f = 0.532 and the minimum accepted power for the test was set at 80% [9]. Primary molars with ICDAS scores of 5 or 6 were collected as they had been indicated for extraction due to caries, orthodontic, or periodontal reasons. The study received ethical approval from the local research ethics committee. Until the time of experiment and during the experiment, the teeth were stored in 37ºC distilled water at incubator (Thermal Laboratory Instruments, Istanbul, Türkiye).

### Procedure

**Fig. 2:**
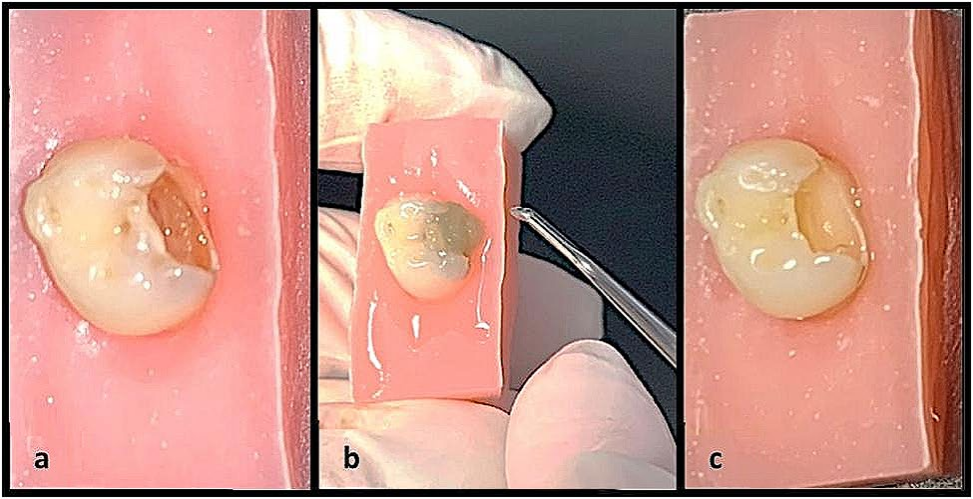
**a**: Initial state, **b**: Application of BRIX3000 and removing carious tissue using excavator, **c**: After caries removal

A chemomechanical caries removal agent, BRIX3000 papain gel (Brix Medical Science, Santa Fe, Argentina) with excavator, was used for removing carious tissue. The gel was applied following the manufacturer’s recommendations, it is allowed to sit for 2 min to soften the carious tissue, facilitating its easy removal with excavator assistance. The stages of caries tissue removal are depicted in Fig. 2.

**Fig. 3:**
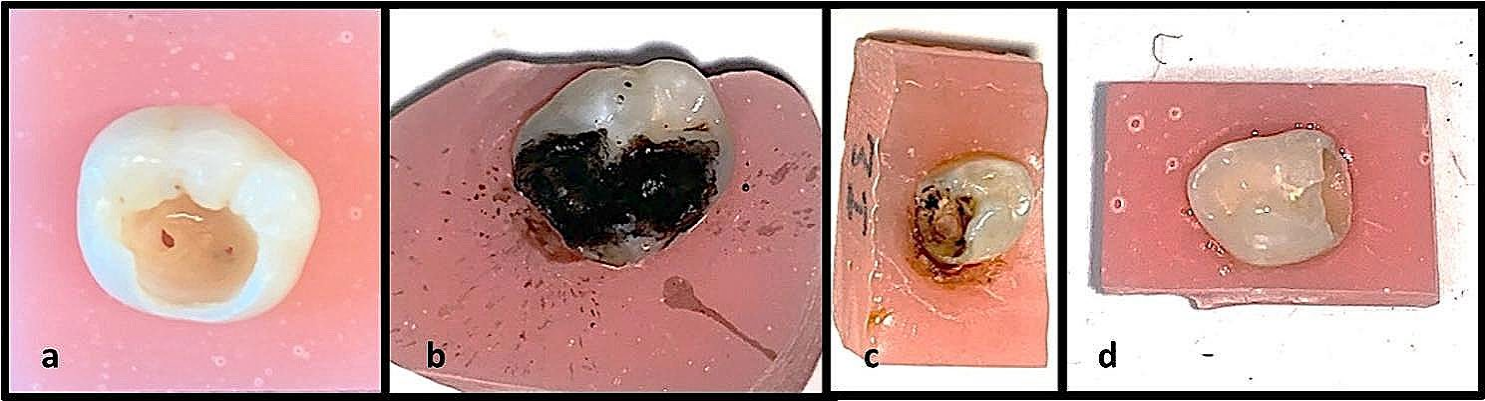
Images immediately after lightcuring of the adesive agent. **a**; Group 1 (control group), **b**; Group 2 (SDF), **c**; Group 3 (SDF/KI), **d**; Group 4 (NSF)

**Fig. 4:**
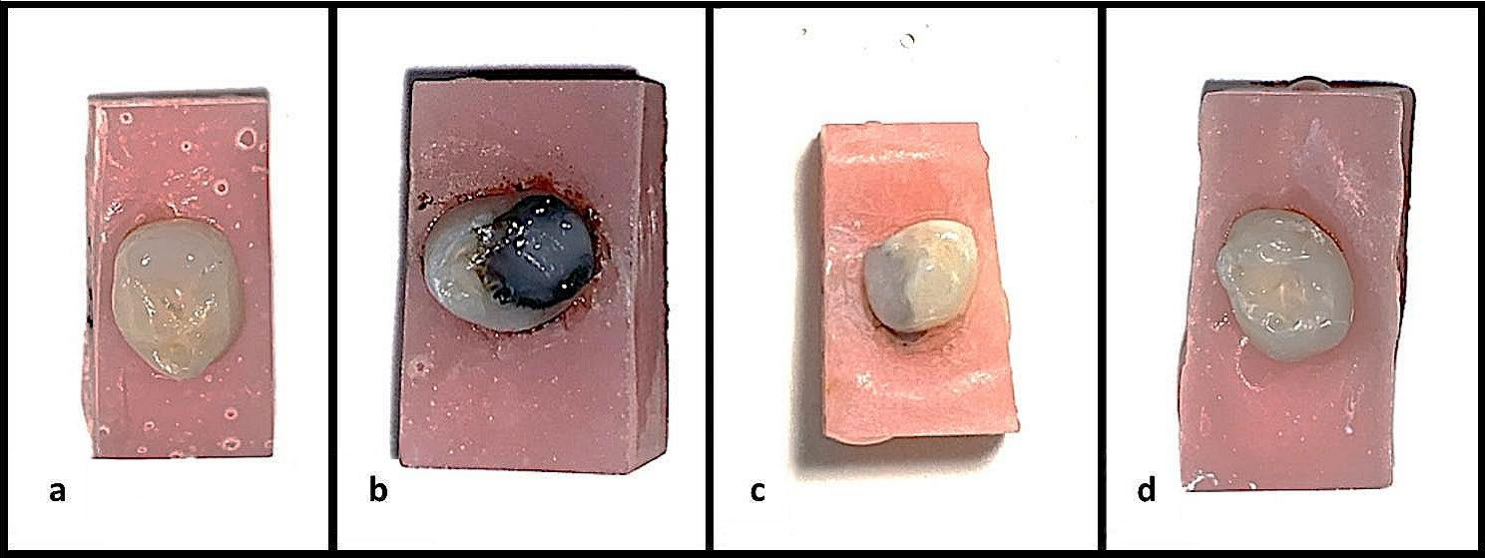
Images immediately after restoration. **a**; Group 1 (control group), **b**; Group 2 (SDF), **c**; Group 3 (SDF/KI), **d**; Group 4 (NSF).

After the chemomechanical caries removal process was completed, the application of the solutions commenced. No solution was applied in Group 1 (control group), while SDF (SDI Riva Star, Victoria, Australia) was applied in Group 2, SDF/KI (SDI Riva Star, Victoria, Australia) in Group 3 and NSF in Group 4. SDF and NSF were applied to the cavity for 1 min with the aid of microbrushes. In Group 3, after SDF application for 1 min, KI was applied until white precipitation disappeared and 30 s of thorough rinsing with copious water. After the application of all solutions was completed, excess solutions were removed with the help of cotton pellets and dried with air for 5 s. Subsequently, filling was performed, and GC Premio Bond (GC Corporation, Tokyo, Japan) was applied following the manufacturer’s recommendations as the adhesive agent. All teeth were restorated with Dyract Xp A2 colored compomer (Denstply Sirona, Bensheim, Germany). After the caries removal process, the filling procedure was directly initiated as no solution was applied in Group 1. Immediate images after light-curing with Elipar DeepCure-S (3 M Oral Care, St. Paul, USA) of the adhesive agent are depicted in Fig. 3, while images immediately after restoration across all groups are presented in Fig. 4.

### Color measurement

Spectrophotometers measure and record the L*, a*, and b* values of an object based on the wavelength of the emitted energy. These values represent the positions of colors on the X, Y, and Z axes and indicate the color coordinates in the CIE L*a*b* color system. The value expressed solely by L* represents the brightness value of a group at a particular time point. The ΔL value, on the other hand, expresses the change in brightness over time and negative value indicates a decrease in brightness, while positive value an increase in brightness, and is obtained by subtracting the value from the first time point from the value from the last time point. The ΔE value, calculated using the L*, a*, and b* values, indicates the amount of color change over a certain period of time and is calculated using the formula ΔE = (ΔL + Δa + Δb)^1/2^ [10].

The color evaluation immediately after restoration (T_0_), after one week (T_1_), and four week later (T_2_), were documented using Vita Easyshade Compact spectrophotometer device (Vita Zahnfabrik, Bad Säckingen, Germany). Values (L*, a* and b*) obtained at three different times were recorded, and changes in color (ΔE) and brightness (ΔL) at intervals T_1_-T_0_ and T_2_-T_0_ were calculated.

### Statistical analysis

The data underwent analysis using IBM SPSS V23 and the R program with the WRS2 package (IBM SPSS Version 23.0. Armonk, NY: IBM Corp). Conformance to a normal distribution was evaluated through the assessment of skewness and kurtosis coefficients within the range of ± 3. Parameters displaying a normal distribution in terms of both group and time were subjected to analysis utilizing Generalized Linear Models, followed by multiple comparisons conducted via the Bonferroni Correction. Parameters that did not adhere to a normal distribution within the context of group and time were subjected to analysis using the Robust ANOVA Test, followed by multiple comparisons with the application of the Bonferroni Correction. Analysis outcomes are presented as mean ± standard deviation and median (minimum–maximum). A significance level of *p* < 0.050 was adopted.

## Results

The group main effect and the time main effect were found to be statistically significant on L* (*p* < 0.001). Over time, a decrease in brightness was observed in all groups. When examining the interaction between group and time, a statistically significant difference in L* was again found (*p* = 0.044). The highest L* of 77.43 was obtained in Group 1 at T_0_. The lowest L* of 55.14 was obtained in Group 2 at T_2_. In Group 3, despite being higher than Group 2, lower L* was observed compared to Group 1 and Group 4 at T_2_. The Partial Eta-Squared value represents the component with the most significant effect. The group and thus the applied solution indicate a greater effect over time on the evaluated value. All values are presented in the following Table 1.

**Table 1:** Descriptive statistics and multiple comparison results of L_0_, L_1_, and L_2_ by group and time

Time		Group				Total
		Group 1 (Control)	Group 2 (SDF)	Group 3 (SDF/KI)	Group 4 (NSF)	
Immediately after (L_0_)		77.43 ± 2.47^A^	61.99 ± 4.62^E^	71.63 ± 3.18^BCD^	74.05 ± 2.94^AB^	71.27 ± 6.67^a^
One week later (L_1_)		77.26 ± 2.5^A^	58.14 ± 5.38^EF^	67.68 ± 2.39^D^	73.88 ± 2.12^AB^	69.24 ± 8.04^b^
Four week later (L_2_)		74.78 ± 2.33^AB^	55.14 ± 4.92^F^	68.5 ± 2.94^CD^	72.95 ± 1.85^ABC^	67.84 ± 8.37^b^
**Total**		76.49 ± 2.66^a^	58.42 ± 5.6^d^	69.27 ± 3.26^c^	73.63 ± 2.33^b^	69.45 ± 7.8
**Group effect**	**F**	186.77				
	***P***	**< 0.001**				
	**η² (Eta-squared)**	0.824				
**Time effect**	**F**	11.79				
	**P**	**< 0.001**				
	**η² (Eta-squared)**	0.164				
Group*Time effect	**F**	2.23				
	**P**	**0.044**				
	**η² (Eta-squared)**	0.101				

Data are expressed as the mean $\pm$ standard deviation

a-d: No significant difference among main effects with the same letter; A-F: No significant difference among interactions with the same letter. Partial Eta-Squared = η² (Eta-squared)

The group main effect (*p* < 0.001) and the time main effect (*p* = 0.042) were found to be statistically significant on ΔL. Upon examining the results, the most significant decrease in brightness was observed in Group 2 (-6.85 ± 4.29) during the T_2_-T_0_ time interval. All values are presented in Table 2.

**Table 2:** Descriptive statistics of ΔL_1_ and ΔL_2_ by group and time

Time		Group				Total
		Group 1 (Control)	Group 2 (SDF)	Group 3 (SDF/KI)	Group 4 (NSF)	
ΔL_1_ (T_1_-T_0_)		-0.17 ± 1.61	-3.85 ± 4.99	-3.95 ± 1.91	-0.16 ± 3.55	-2.03 ± 3.71
ΔL_2_ (T_2_-T_0_)		-2.65 ± 1.01	-6.85 ± 4.29	-3.13 ± 1.92	-1.1 ± 3.71	-3.43 ± 3.63
**Total**		-1.41 ± 1.82^bc^	-5.35 ± 4.8^a^	-3.54 ± 1.91^ab^	-0.63 ± 3.58^c^	-2.73 ± 3.71
**Group effect**	**F**	9.94				
	***P***	**< 0.001**				
	**η² (Eta-squared)**	0.271’				
**Time effect**	**F**	4.27				
	**P**	**0.042**				
	**η² (Eta-squared)**	0.051				
Group*Time effect	**F**	1.61				
	**P**	0.193				
	**η² (Eta-squared)**	0.057				

Data are expressed as the mean $\pm$ standard deviation

a-c: No significant difference among main effects with the same letter. Partial Eta-Squared = η² (Eta-squared)

The group main effect (*p* = 0.013) and the time main effect (*p* = 0.019) were found to be statistically significant on ΔE. The highest color change was observed in Group 2 (9.52), while over the long term, SDF/KI and NSF caused similar levels of staining during the T_2_-T_0_ time interval. All values are presented in the following Table 3.

**Table 3:** Descriptive statistics of ΔE_1_ and ΔE_2_ by group and time

Time		Group				Total
		Group 1 (Control)	Group 2 (SDF)	Group 3 (SDF/KI)	Group 4 (NSF)	
ΔE_1_ (T_1_-T_0_)		2.74 (1.15–8.07)	5.1 (1.91–11.18)	4.78 (1.43–11.4)	3.68 (1.56–5.84)	4.01 (1.15–11.4)
ΔE_2_ (T_2_-T_0_)		4.15 (1.58–7.13)	9.52 (3.33–14.9)	4.74 (1.72–8.59)	4.95 (1.21–7.79)	5.05 (1.21–14.9)
**Total**		3.54 (1.15–8.07)^b^	6.99 (1.91–14.9)^a^	4.76 (1.43–11.4)^ab^	3.91 (1.21–7.79)^ab^	4.63 (1.15–14.9)
**Group effect**	**Q**	3.567				
	***P***	**0.013**				
**Time effect**	**Q**	5.497				
	**P**	**0.019**				
Group*Time effect	**Q**	3.412				
	**P**	0.332				

Data are expressed as the mean $\pm$ standard deviation

a-b: No significant difference among main effects with the same letter. Q: Robust ANOVA Test Statistics

## Discussion

Numerous studies have been conducted on the discoloration effect of SDF [4, 5, 9]. However, the number of studies investigating the discoloration effect of SDF/KI and NSF on dental tissues is quite limited in the literature. This study is the first to investigate and compare the discoloration effect of SDF, SDF/KI, and NSF solutions on commonly used compomer filling material in pediatric dentistry.

In accordance with the findings of the systematic review published by Roberts et al., it is established that application of KI after SDF offers advantages in diminishing staining [11]. Fröhlic et al. [12] examined the impact of KI on staining by partitioning 30 dentin specimens derived from bovine teeth into three groups following demineralization. Group 1 underwent no treatment, Group 2 received SDF, and Group 3 was treated with SDF/KI. The findings demonstrated that KI application reduced staining. Nguyen et al. [13] conducted an in vitro study to investigate the staining efficacy of a 10% KI solution on various restoration materials. Teeth that were treated with SDF and then subjected to light-curing exhibited immediate grayish staining. All groups treated with SDF/KI showed similar color change to the control groups, indicating that it was effective in lessening the staining. According to our study, after one week of measurements, it was observed that there was a similar decrease in brightness and color change between SDF/KI and SDF groups. However, it was found that it caused a greater decrease in brightness and color change compared to the control and NSF groups. After four weeks, although the use of SDF/KI was found to be advantageous compared to SDF, it was observed that it did not completely prevent color change compared to the control group.

Zhao et al. [14] assessed the staining effect of SDF and SDF/KI on glass ionomer restoration margins in their in vitro study. Pairwise comparisons reveal that SDF/KI induces less staining compared to SDF, although KI does not completely eliminate the staining effect of SDF at the restoration margins. Miller et al. [15] conducted an in vitro study using a subjective scale ranging 0–5, focusing on the preventive impact of KI on staining of glass ionomer. In contrast, no significant difference in staining were found between SDF and SDF/KI treated groups. In our study, it was found that KI did not completely prevent the black staining effect of SDF, but it could be advantageous compared to SDF in the long term. However, the use of only compomer is one of the limitations of the study. In future research, it is believed that comparing the effect of SDF, SDF/KI, and NSF solutions on staining using a non-light-curing filling material alongside light-curing ones could be beneficial in understanding the effect of light-curing.

Kamble et al. [9] collected 30 carious primary molars and carried out various restoration applications. They treated with SDF in Group 1, SDF/KI in Group 2, and SDF + Glutathione (GSH) in Group 3 followed by restoration with glass ionomer. Color evaluations were conducted using spectrophotometer. Measurements taken at the end of day 1, 1 week, and 4 weeks revealed the highest ΔE values and staining in SDF. The study underlined the need for further research in this area while highlighting the potential advantages of KI and GSH in decreasing staining. In our study, GSH was excluded due to the increase in sample size and costs. However, it is believed that future research would benefit from understanding whether the combination of SDF with KI or GSH is more advantageous and comparing the staining effect with NSF solution.

Hamdy et al. [16] conducted an in vitro study, in which they carried out color assessments using spectrophotometer. Initial measurements were recorded before the experimental materials were applied. SDF, SDF + composite, SDF/KI, and SDF + glass ionomer was performed in groups respectively. Second color measurements were taken immediately after the application of these materials, and third color measurements were taken after 24-hour suntest aging protocol. In the SDF group, noticeable decrease in L*, a*, and b* values over time was observed. It was reported that SDF caused significant black staining in the carious dentin, and the restorative material and KI could reduce the staining effect of SDF. The masking effect of the composite was noted to be the most successful material that was not fully reversed by the aging procedure. Another limitation of our study is that we only conducted color measurements after applying the filling material. Considering the potentially masking effect of the filling material’s black staining, the obtained values may not reflect the true black staining effects of the solutions. In future research, it would be beneficial to follow staining both with and without applying the filling material after the application of the solutions to compare the results.

There is a limited amount of research in the literature that examines the staining-inducing effect of NSF on teeth and restorative materials. Therefore, our study aimed to explore whether NSF causes staining on filling materials. The formation of oxides is not observed when nanosilver particles come into contact with teeth. Therefore, it is noted that NSF does not cause staining on teeth, but slight level of staining may occur in those with nanoparticle sizes of approximately 100 nm [17].

Espíndola-Castro et al. [18] examined the color changes that occurred following the application of NSF formulations with 600 and 1500 ppm concentrations on dentin samples compared to SDF and SDF/KI. The assessments were conducted using spectrophotometer immediately after application, 2 weeks, and 4 weeks. The samples were stored in artificial saliva until the end of the experimental period. At the end of 4 weeks, before the color measurement process, the samples were brushed without the use of any abrasive materials. The results revealed that the NSF formulations resulted in less staining compared to other groups. The yellowish stains caused by NSF led to decrease in brightness at the measurement after 2 weeks, but it was observed that the brushing process could remove the stains. In their in vitro study conducted on dentin samples obtained from bovine teeth, Sayed et al. [17] examined SDF, AgNPs (Silver Nanoparticles), KF (Potassium Fluoride), and AgNPs/KF applications and conducted color measurements before treatment, immediately after, and at the 1st, 2nd, and 7th days, and analyzed ΔE. They found that SDF exhibited significantly higher staining compared to other groups, while the materials present in the other groups did not cause significant staining. Zhao et al. [19] produced PEG (Polyethylene Glycol)-AgNPs solution containing 11.600 ppm fluoride and 400 ppm silver, with silver nanoparticle size of 2.56 ± 0.43, and conducted color assessments. They found no significant difference in terms of staining between water and PEG-AgNPs solution. The L*, C*, and h* values in the SDF group were notably lower than in the other two groups, and indicating a substantial color change compared to the others. Therefore, it was concluded that PEG-AgNPs solution could serve as a promising alternative to counter the drawback of black staining caused by SDF. Similarly in our study, NSF caused significantly less color change compared to SDF and SDF/KI. However, the limitation of our study lies in the nanoparticles not being coated with PEG. Comparing the staining effect of NSF solutions with both features would be beneficial for future research.

There is a need for further research on materials that can reduce the staining effect of SDF while not compromising its caries-inhibiting, remineralizing, and antibacterial properties or providing alternatives. Ongoing studies focus on the use of KI, GSH, their combination with restorative materials, and determining which content of NSF yields better results. In recent biomedical research, attention has turned to selenium nanoparticles (SeNPs), which are reported to possess antimicrobial, antioxidant, and antiviral capabilities, and investigations are underway in this area [20].

The environment in which teeth are stored during the period of color assessment are crucial. Various methods such as thermal cycling, UV light exposure, incubation in distilled water, storage in phosphate buffered saline, or artificial saliva can be utilized in studies. Distilled water containing NaHCO_3_ and KCl, due to its chemical composition resembling human saliva, can serve as an alternative to artificial saliva in research [21]. Some researchers suggest storing the samples in a dry environment to prevent undesirable reactions that could arise from the interaction between SDF and the storage conditions, and to accurately assess the absolute effect of SDF [17]. Patel et al. [22] have noted that SDF reacts with sodium chloride present in artificial saliva, resulting in the formation of insoluble white silver chloride, which could potentially have a negative impact on color measurements. The use of artificial saliva, which is closer in composition to the oral environment due to its enzymatic content, could provide results that are more realistic. However, researchers have indicated that it might reduce the material’s staining effect and that the clear color change effect could be better observed in a dry environment because there is no risk of the staining effect being removed by liquid’s removal effect. Hence, it is considered beneficial for future studies to compare color changes in different storage environments.

## Conclusions

In conclusion, this study indicates that SDF/KI and NSF solutions are found to be advantageous over SDF in terms of the black staining effect in long term; however, more studies are needed to support their usage further.

## Data Availability

The datasets used and/or analysed during the current study available from the corresponding author on reasonable request.

## References

[1] Contreras V, Toro MJ, Elías-Boneta AR, Encarnación-Burgos A (2017). Effectiveness of silver diamine fluoride in caries prevention and arrest: a systematic literature review. Gen Dent.

[2] Seifo N, Robertson M, MacLean J, Blain K, Grosse S, Milne R, Seeballuck C, Innes N. The use of silver diamine fluoride (SDF) in dental practice. Br Dent J. 2020;228(2):75–81. 10.1038/s41415-020-1203-9. PMID: 31980777. 10.1038/s41415-020-1203-9 31980777

[3] Yan IG, Zheng FM, Gao SS, Duangthip D, Lo ECM, Chu CH (2022). Ion Concentration of Silver Diamine Fluoride Solutions. Int Dent J.

[4] Turton B, Horn R, Durward C (2021). Caries arrest and lesion appearance using two different silver fluoride therapies on primary teeth with and without potassium iodide: 12-month results. Clin Exp Dent Res.

[5] Aly MM, Yousry YM. Potential discolouration of silver diamine fluoride versus silver diamine fluoride/potassium iodide in primary teeth: a randomised clinical study. Br Dent J. 2022;1–6. 10.1038/s41415-022-5272-9. PMID: 36473976; PMCID: PMC9734755. 10.1038/s41415-022-5272-9 PMC9734755 36473976

[6] Khubchandani M, Thosar NR, Dangore-Khasbage S, Srivastava R (2022). Applications of silver nanoparticles in Pediatric Dentistry: an overview. Cureus.

[7] Favaro JC, Detomini TR, Maia LP, Poli RC, Guiraldo RD, Lopes MB, Berger SB (2022). Anticaries Agent based on silver nanoparticles and fluoride: characterization and Biological and Remineralizing Effects-An in Vitro Study. Int J Dent.

[8] Pushpalatha C, Bharkhavy KV, Shakir A, Augustine D, Sowmya SV, Bahammam HA, Bahammam SA, Mohammad Albar NH, Zidane B, Patil S (2022). The anticariogenic efficacy of Nano Silver Fluoride. Front Bioeng Biotechnol.

[9] Kamble AN, Chimata VK, Katge FA, Nanavati KK, Shetty SK (2021). Comparative evaluation of Effect of Potassium Iodide and glutathione on tooth discoloration after application of 38% silver diamine fluoride in primary molars: an in Vitro Study. Int J Clin Pediatr Dent.

[10] Chang JY, Chen WC, Huang TK, Wang JC, Fu PS, Chen JH (2015). Evaluation of the accuracy and limitations of three tooth-color measuring machines. J Dentistry Sci.

[11] Roberts A, Bradley J, Merkley S, Pachal T, Gopal JV, Sharma D. Does potassium iodide application following silver diamine fluoride reduce staining of tooth? A systematic review. Aust Dent J. 2020;65(2):109–17. 10.1111/adj.12743. PMID: 31900927. 10.1111/adj.12743 31900927

[12] Fröhlich TT, Gindri LD, Pedrotti D, Cavalheiro CP, Soares FZM, Rocha RO (2021). Evaluation of the Use of Potassium Iodide Application on stained demineralized dentin under Resin Composite following silver diamine fluoride application. Pediatr Dent.

[13] Nguyen V, Neill C, Felsenfeld J, Primus C (2017). Potassium iodide. The solution to silver diamine fluoride discoloration?. Adv Dent Oral Health.

[14] Zhao IS, Mei ML, Burrow MF, Lo EC, Chu CH (2017). Effect of silver diamine fluoride and potassium iodide treatment on Secondary Caries Prevention and Tooth Discolouration in Cervical Glass Ionomer Cement Restoration. Int J Mol Sci.

[15] Miller MB, López LA, Quock RL (2016). Silver diamine fluoride, potassium iodide, and esthetic perception: an in vitro pilot study. Am J Dent.

[16] Hamdy D, Giraki M, Abd Elaziz A, Badran A, Allam G, Ruettermann S (2021). Laboratory evaluation of the potential masking of color changes produced by silver diamine fluoride in primary molars. BMC Oral Health.

[17] Sayed M, Hiraishi N, Matin K, Abdou A, Burrow MF, Tagami J. Effect of silver-containing agents on the ultra-structural morphology of dentinal collagen. Dent Mater. 2020;36(7):936–44. doi: 10.1016/j.dental.2020.04.028. PMID: 32475750. 10.1016/j.dental.2020.04.028 32475750

[18] Espíndola-Castro LF, Rosenblatt A, Galembeck A, Monteiro G. Dentin Staining Caused by Nano-silver Fluoride: A Comparative Study. Oper Dent. 2020;45(4):435–41. 10.2341/19-109-L. PMID: 32053463. 10.2341/19-109-L 32053463

[19] Zhao IS, Yin IX, Mei ML, Lo ECM, Tang J, Li Q, So LY, Chu CH (2020). Remineralising dentine caries using Sodium Fluoride with Silver nanoparticles: an in Vitro Study. Int J Nanomed.

[20] Almuqrin A, Kaur IP, Walsh LJ, Seneviratne CJ, Zafar S (2023). Amelioration strategies for silver diamine fluoride: moving from Black to White. Antibiot (Basel).

[21] Baines S, Hensels IS, Talmi D. The use of ‘artificial saliva’ as a neutral control condition in gustatory research: Artificial saliva is not a neutral gustatory stimulus. Physiol Behav. 2021;229:113254. 10.1016/j.physbeh.2020.113254. PMID: 33220327. 10.1016/j.physbeh.2020.113254 33220327

[22] Patel J, Anthonappa RP, King NM. Evaluation of the staining potential of silver diamine fluoride: in vitro. Int J Paediatr Dent. 2018;28(5):511–22. 10.1111/ipd.12401. PMID: 29974546. 10.1111/ipd.12401 29974546

